# Effects of Non-invasive Brain Stimulation on Stimulant Craving in Users of Cocaine, Amphetamine, or Methamphetamine: A Systematic Review and Meta-Analysis

**DOI:** 10.3389/fnins.2019.01095

**Published:** 2019-10-18

**Authors:** Tianye Ma, Yurong Sun, Yixuan Ku

**Affiliations:** ^1^The Shanghai Key Lab of Brain Functional Genomics, Shanghai Changning-ECNU Mental Health Center, School of Psychology and Cognitive Science, East China Normal University, Shanghai, China; ^2^School of Medicine, Tongji Hospital, Tongji University, Shanghai, China; ^3^College of Psychology and Sociology, Shenzhen University, Shenzhen, China; ^4^NYU Shanghai and Collaborative Innovation Center for Brain Science, NYU-ECNU Institute of Brain and Cognitive Science, Shanghai, China

**Keywords:** non-invasive brain stimulation, addiction, substance use disorders, dopamine system, craving

## Abstract

Dopamine system plays a pivotal role in specific kinds of substance use disorders (SUD, i. e., cocaine and methamphetamine use disorders). Many studies addressed whether dopamine-involved craving could be alleviated by non-invasive brain stimulation (NIBS) techniques. Nevertheless, the outcomes were highly inconsistent and the stimulating parameters were highly variable. In the current study, we ran a meta-analysis to identify an overall effect size of NIBS and try to find stimulating parameters of special note. We primarily find 2,530 unduplicated studies in PubMed, Psychology and Behavioral Sciences Collection, PsycARTICLES, PsycINFO, and Google Scholar database involving “Cocaine”/“Amphetamine”/“Methamphetamine” binded with “TMS”/“tDCS”/“non-invasive stimulation” in either field. After visual screening, 26 studies remained. While 16 studies were further excluded due to the lack of data, invalid craving scoring or the absence of sham condition. At last, 16 units of analysis in 12 eligible studies were coded and forwarded to a random-effect analysis. The results showed a large positive main effect of stimulation (Hedge's *g* = 1.116, CI = [0.597, 1.634]). Further subgroup analysis found that only high-frequency repetitive transcranial magnetic stimulation (rTMS) could elicit a significant decrease in craving, while the outcome of low-frequency stimulation was relatively controversial. Moreover, univariate meta regression revealed that the number of pulses per session could impose negative moderation toward the intervention. No significant moderation effect was found in types of abuse, overall days of stimulation and other variables of stimulating protocol. In conclusion, this meta-analysis offered a persuasive evidence for the feasibility of using NIBS to remit substance addictive behavior directly based on dopamine system. We also give clear methodological guidance that researchers are expected to use high-frequency, sufficiently segmented rTMS to improve the efficacy in future treatments.

## Introduction

Drug addiction, also known as substance use disorder (SUD), is a severe threat to physical and psychological health, which is suffered by at least 275 millions of people all over the world. This medical situation is defined as the compulsive active use of substances regardless of the potential harms and recruits a series of diagnosis criteria including withdrawal symptoms, craving, physical and mental illness, etc. (American Psychiatric Association, 2013). Addiction to certain kinds of substances has also been found to negatively impact working memory (Yan et al., 2014), response inhibition (Goldstein et al., 2001), emotional empathy (Ferrari et al., 2014), and decision making (Bechara et al., 2001). Hence, unraveling the mechanism of SUD and inventing effective treatments have always been the pivotal goals in neuroscience studies.

Most kinds of SUDs are generally considered to originate from abnormality in dopamine (DA) system (except for opioid and cannabis addiction, see Nutt et al., 2015 for review). Stimuli such as drugs or predictive cues of drugs modulate the firing pattern of dopaminergic neurons in ventral tegmental area (VTA) and elicit a large DA release which represents the reward prediction error (Schultz, 2002). The signal will be projected to GABAergic medium spiny neurons (MSNs) expressing DA receptors in the nucleus accumbens (NAc) of ventral striatum (Paladini and Roeper, 2014; Volkow and Morales, 2015). Weights of connections between MSNs and cortical areas could then be altered. A bunch of imaging studies have revealed that the repeated use of cocaine and amphetamine-like substances will downregulate DA release and DA receptor availability (Ashok et al., 2017) which results in the attenuation of projections to the cortical areas such as the dorsolateral prefrontal cortex (DLPFC), anterior cingulate cortex (ACC), and orbitofrontal cortex (OFC) (Black et al., 2010; Volkow et al., 2011). These targeted regions are responsible for executive control functions or salience attribution to the external stimuli (Fuster, 2015). This might explain why the abusers are hardly able to control the craving and consumption of drugs. In general, the dysfunction of dopamine pathway plays a central role in drug addiction and this notion has inspired the development of neurobiological treatments including acupuncture (Lee et al., 2009), pharmacotherapies (Lu et al., 2009), neurosurgical operations (Stelten et al., 2008), and brain stimulations (Müller et al., 2013; Hanlon et al., 2016).

## Non-Invasive Brain Stimulation as a Potential Treatment for SUD

Although previous studies have constructed relatively thorough understandings toward the brain mechanism of SUD, we still haven't found credible and efficient ways of treatment yet. However, NIBS seemingly gave us a new direction in the recent years. Transcranial magnetic stimulation (TMS) is one typical NIBS technique. It applies magnetic pulses to a certain location on the scalp to induce spike firing in the corresponding brain tissue. Single-pulse TMS has been proved to produce changes in many aspects including visual perception (Van Ettinger-Veenstra et al., 2009), working memory (Ku et al., 2015a,b; Zhao and Ku, 2018; Zhao et al., 2018), motor learning (Bütefisch et al., 2004), interpersonal cooperation (Balconi and Canavesio, 2014), etc. While repetitive TMS (rTMS), which employs multiple trains of pulses within a single block, is more suitable for therapeutic purposes. A common belief is that high-frequency (5–20 Hz) rTMS elicits cortical excitation while low-frequency (~1 Hz) pulses conversely lead to inhibition. However, there are still exceptions that make the relationship between the stimulating parameter and the subsequent cortical effect controversial (Paus et al., 1998). Besides, the counterpart of TMS, transcranial direct current stimulation (tDCS), which also has broad applications in cognitive enhancement and treatments (Sauvaget et al., 2015; Wang and Ku, 2018; Wang et al., 2019), modulates neural activity by directly imposing current flow into the brain between two electrode patches. Nitsche and Paulus (2000) find that the anode tDCS could increase excitability in motor areas, while cathodal tDCS induces inhibition. However, more studies are needed to test whether this conclusion is robust across different sets of stimulating parameters and whether the activation could transfer to other non-stimulating brain areas as well.

Several clinical trials have reported alleviation of stimulant craving of NIBS compared to control group (Bolloni et al., 2016; Hanlon et al., 2017; Liu et al., 2019). Most of them choose the DLPFC as a stimulating site in the light of the notion that this region is important for executive control. Martinez et al. (2018) recruit Hedes-coil (H-coil) to stimulate deep brain regions (ACC and medial PFC) of analogous functions in the dopamine pathway and find significant alleviation in cocaine craving when stimulating frequency is set to 10 Hz. In line with the conventional view, low-frequency rTMS or continuous theta-burst stimulation (cTBS) does not change the level of craving or even boosts craving in most occasions (Li et al., 2013; Hanlon et al., 2017; Martinez et al., 2018). Nonetheless, Liu et al. (2017) find inconsistent results regarding this issue in a group of methamphetamine abusers. The existing studies also have prominent discrepancies in parameters such as overall days of stimulation, number of sessions, number of pulses other than rTMS frequency. Given these controversial issues, a comprehensive analysis will be fruitful in the development of a more effective and reliable treatment protocol.

Jansen et al. (2013) run a meta-analysis for the potential effect of NIBS toward DLPFC on craving for food or stimulants, and find a medium treatment effect (Hedge's *g* = 0.476, CI = [0.316, 0.636]). Gorelick et al. (2014) separate several groups of independent meta-analysis for each kinds of stimulants and all the results suggest significant decrease in craving. However, these two studies do not discuss the optimal stimulating protocol quantitatively. Furthermore, although Song et al. (2019) test the relationship between stimulating parameters and the outcome of NIBS, they combine the results from SUD, eating disorder, and obesity. It might not be tenable to apply these results to SUD treatment precisely. Thus, in the current study, we take a re-consideration toward the role of NIBS in the treatment of SUD by implementing a meta-analysis which focuses on the prospective modulators that might be of special importance to the stimulating protocol.

Additionally, we only include studies of cocaine, amphetamine and methamphetamine addiction as they are substances that act directly on DA receptors. TMS on rats' frontal cortices could induce DA release (Zangen and Hyodo, 2002). Deep rTMS of human studies reveals similar effects (Ceccanti et al., 2015). Likewise, DA transporter availability in caudate nucleus goes up after a high-frequency rTMS on DLPFC in a recent case study (Pettorruso et al., 2019). Moreover, tDCS on bilateral DLPFC elicits DA increase in the same region as well (Fonteneau et al., 2018). Put all these findings together, the treatment effect of NIBS is possibly derived from the alteration of DA level through the feedback pathway from frontal cortices to striatum (Diana, 2011, Figure 1). By prescribing the three types of addiction in the current study, we aim to call the attention to this DA theory of NIBS treatment.

**Figure 1:**
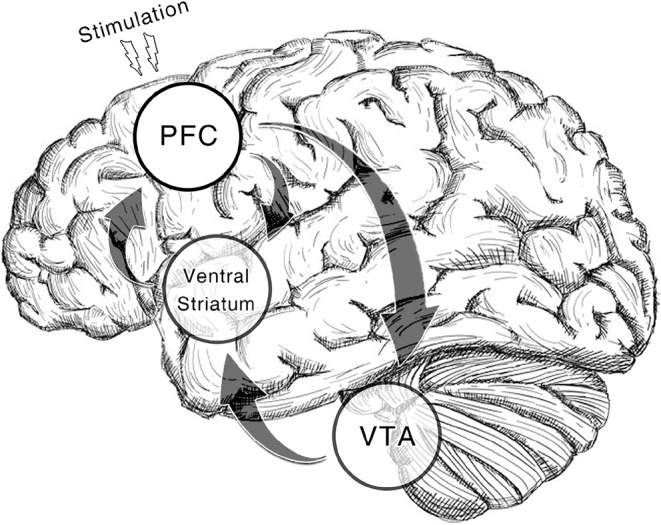
Dopamine reward system involved in the therapeutic effect of NIBS. In the illustrated pathway, dopaminergic neurons in ventral tegmental area (VTA) projects the reward signal to medium spiny neurons (MSNs) in ventral striatum by which the cortico-striatal connection is modulated. While prefrontal regions (pyramidal neurons) including dorsolateral prefrontal cortex (DLPFC), anterior cingulate cortex (ACC), and medial prefrontal cortex (mPFC) give feedback to these regions (Gorelova and Yang, 1996; Frankle et al., 2006). With non-invasive brain stimulation alters activation in prefrontal regions, the VTA reactivity will be enhanced which results in the recovery of DA increase in the downstream areas. The regions with a transparent circle rearward are not on the cortical surface.

## Materials and Methods

### Study Inclusion Criteria

For the homogeneity and validity of our meta-analysis, we set a few ex ante principles to filter the studies based on the theoretical background.

#### NIBS Treatment

Qualified studies should employ NIBS as the only method of treatment and report whether it alleviates craving. Deep brain stimulation and other kinds of treatments are expected to be excluded. A study used 5 Hz cTBS (Hanlon et al., 2017) that is also regarded as rTMS, is included in our analysis, whereas it does not join in the meta-regression of stimulation frequency.

#### Type of Addiction

As previously mentioned, only the trials targeted at cocaine and amphetamine-like drug addiction will be included. Thus, studies with whom take opioid, cannabis, tobacco, alcohol, food abusers, or are non-abusers as participants are invalid. Mixed abuse shall be acceptable as long as the study probed the alteration of craving toward the drug of our interest.

#### Sham Comparison

Control strategy is necessary in order to rule out the impact of placebo effect. Groups should be randomly assigned. Moreover, sham stimulation is the only valid way of control since the difference between abusers and normal subjects could be possibly attributed to the floor effect of craving in the normal group. Within-group comparison between separated sham and stimulation sessions is also qualified.

#### Indicators of Craving

Clinical trials have used different methods to acquire craving scores. While in the current analysis, the studies shall not be restricted by the methodology of craving assessment only if the indicator itself could not directly represent the level of craving such as the amplitude of cue-induced event-related potential (Conti and Nakamura-Palacios, 2014; Conti et al., 2014). Bolloni et al. (2016) applied the quantity of cocaine residue in hair samples to indicate craving. Their study is also included in the analysis as cocaine intake is motivated by the underlying desire, so it should be proportional to the level of craving.

### Search Strategy and Study Selection

**Figure 2:**
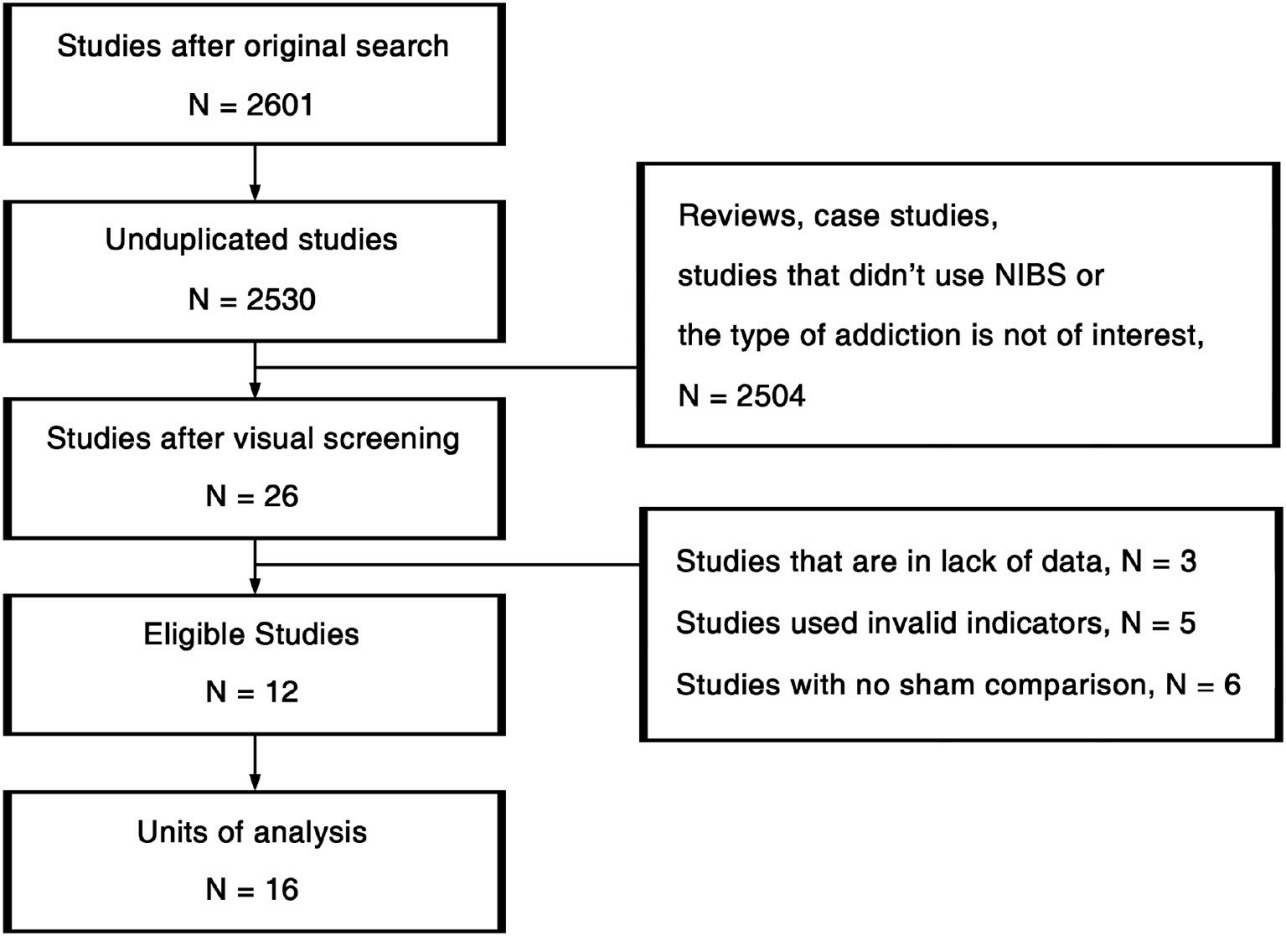
Flow diagram of the study selection procedure.

The procedures of study selection are annotated in Figure 2. We used 3-by-3 keywords composed by “Cocaine” /“Amphetamine”/“Methamphetamine” and “Transcranial Magnetic Stimulation”/“Transcranial Direct Current Stimulation”/“Non-Invasive Brain Stimulation” (NIBS) in the search across PubMed, Psychology & Behavioral Sciences Collection, PsycARTICLES, PsycINFO, and Google Scholar. All the studies detected in the original search were first unduplicated. Afterwards, the remaining 2,530 studies were visually screened based on titles and abstracts. Then we read the full texts of the 26 studies passed the initial screening. Eleven studies that did not fit the inclusion criteria, 3 studies that were in lack of data were further excluded. At last, 12 eligible studies were viewed again for data extraction.

### Data Extraction

Different sessions of the same group of participants that employed different stimulating parameters were treated as independent units of design. Consequently, we detected 16 units of analysis in those 12 studies, covering 321 patients altogether. Given that pretest craving scores of the control group and the stimulating group did not have significant difference in all of the included studies, the therapeutic effect of each unit was coded as the difference of craving in the posttest that was closest to the end of treatment in terms of time. Subjects' demographics including gender, age, years of education, duration of addiction, and duration of abstinence in each group were extracted. Only gender, age, duration of addiction were coded as potential modulators and forwarded to meta-regression while other variables were lack of detailed information. We coded the stimulation method as “tDCS” or “rTMS.” Given that there were only two units of analysis using tDCS, we only discussed the protocol of rTMS in the current study. Overall, categorical variables including sites of stimulation, types of addiction, rTMS frequency (1 Hz or larger than 5 Hz) and continuous variables including sessions, days, pulses, pulses per session of rTMS treatment were further extracted as possible modulators. Results of coding and other information regarding each study are summarized in the Supplementary Material (Data Sheets 1, 2). Note that among the 12 eligible trials, none of them were about amphetamine addiction, so the following analysis was merely about the existing methamphetamine and cocaine studies.

### Data Analysis

All of our analysis was done in Comprehensive Meta Analysis V2 (Borenstein et al., 2009). Given that the sample sizes of the included studies are basically small, we used Hedge's *g* to calculate the effect size which can rectify the bias induced by small samples (Hedges, 1981).

We first estimated the overall effect size of the NIBS's therapeutic effect using a random-effect model which assumed that the observed effect size in each study was a combination of the true effect size sampled from an underlying normal distribution and a random error. The reason for choosing this model is that the effect was expected to vary according to the hypothesized modulation by stimulating frequency and other factors. The heterogeneity between studies was assessed by Cochrane's *Q* and *I*^2^ value. To test the modulators, we employed subgroup analysis using mixed-effect model and fixed-effect univariate meta-regression for categorical and continuous variables, respectively. Significant level was designated as 0.05 in all analyses.

## Results

### Therapeutic Effect of NIBS

**Figure 3:**
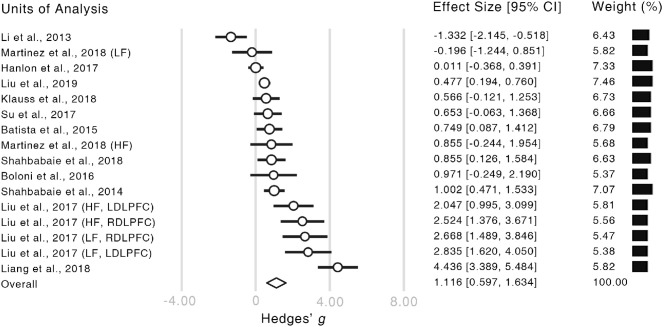
Forest plot of the estimated effect sizes. Authors and years of publication of each unit of analysis are shown in the very left column. The words in the brackets indicate the information of within-study subgroups (HF, high frequency; LF, low frequency; LDLPFC, left dorsolateral prefrontal cortex; RDLPFC, right dorsolateral prefrontal cortex). In the forest plot, the length of each bar illustrates the confidence interval of the corresponding unit. The size of each empty circle illustrates the relative weight of each unit which is also showed by the black bars on the right-hand side.

The meta-analysis revealed a significantly strong effect of NIBS on the alleviation of craving levels (Hedge's *g* = 1.116, CI = [0.597, 1.634], *z* = 4.218, *p* < 0.001, Figure 3). Moreover, both of the Rosenthal's (1979) and Orwin's (1983) fail-safe *N* proved the credibility of our result (Rosenthal's *N* = 399, Orwin's *N* = 76). Given that the number of studies in our analysis was relatively small, the resultant effect size could not be fully explained by publication bias (Ridding and Rothwell, 2007).

### Heterogeneity Across Studies

As predicted, heterogeneity among the observed effect sizes was significant (*I*^2^ = 88.548%, *Q* = 130.978, *p* < 0.001) which indicated the between-study variation could not merely be attributed to the random error. Thus, we then traced the possible origin of the heterogeneity by testing the possible modulators.

### Modulators

#### Demographic Variables and Duration of Addiction

We first filtered the studies that did not report enough information for each modulator and the number of remaining studies is then denoted by *N*. To assess the relationship between the therapeutic effect and the subject variables, we converted the means of age, gender (percentage of males), and duration of addiction in treatment and control group into between-group difference (treatment—control) or across-group average weighted by group sizes. Meta-regression revealed that age difference (*N* = 14) was negatively correlated with the NIBS effect [*Q*(1) = 54.04, *p* < 0.001], while the weighted average (*N* = 13) had null effect [*Q*(1) = 1.10, *p* = 0.29]. As for gender, between-group discrepancy (*N* = 10) could not predict the effect of NIBS treatment [*Q*(1) = 0.50, *p* = 0.48] whereas weighted average revealed a significant positive modulation effect [*N* = 10, *Q*(1) = 7.15, *p* = 0.008]. The regression between group-wise difference in subjects' years of drug use (*N* = 9) revealed a prominent positive relationship [*Q*(1) = 14.48, *p* < 0.001]. However, the weighted average (*N* = 8) showed a significant converse effect [*Q*(1) = 7.60, *p* = 0.006].

#### Type of Addiction

The mixed-effect subgroup analysis suggested that the treatment for cocaine addiction (*N* = 6, Hedges' *g* = 0.397, CI = [0.022, 0.772], *z* = 2.075, *p* = 0.038) and methamphetamine addiction (*N* = 10, Hedges' *g* = 1.541, CI = [0.735, 2.347], *z* = 3.749, *p* < 0.001) were both effective. There also existed significant difference between the studies of these two kinds of addiction [*Q*(1) = 10.974, *p* = 0.001].

#### Type of Stimulation

As there were only four tDCS studies included in our analysis, we picked out studies only applying rTMS and found that there still existed an overall significant effect (*N* = 12, Hedges' *g* = 1.264, CI = [0.540, 1.989], *z* = 3.419, *p* = 0.001).

#### Stimulating Protocol

We then looked at the relationship between NIBS effect and the stimulating parameters. In the studies using high-frequency rTMS (*N* = 7), the craving level did decrease (Hedges' *g* = 1.671, CI = [0.669, 2.673], *z* = 3.269, *p* = 0.001), while there was no such effect in low-frequency rTMS studies (*N* = 4, Hedges' *g* = 0.962, CI = [−1.137, 3.061], *z* = 0.898, *p* = 0.369), though the low-frequency effect did not significantly differ from the high-frequency effect [*Q*(1) = 2.50, *p* = 0.113]. Although the studies employed different sites of stimulation, we only analyzed the overall effect size of stimulating the left DLPFC (*N* = 6, Hedges' *g* = 1.465, CI = [0.170, 2.760], *z* = 2.217, *p* = 0.027) due to lack of studies in other sites (see Appendix).

**Figure 4:**
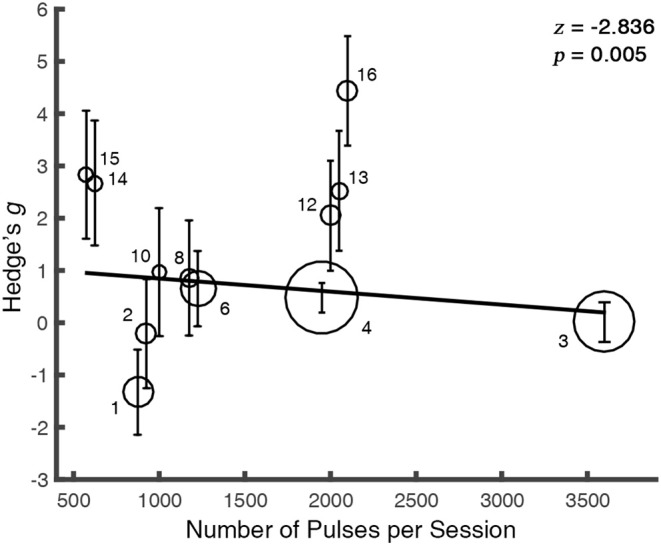
The therapeutic outcome is modulated by the number of pulses per session. For clarity, the overlapped values of regressor were randomly jittered. The solid line is the fixed-effect regressive function for the regressor. The size of each bubble represents the study's relative weight which was calculated as the inverse of the variance. Error bars indicate the 95% CIs. The markers abut to the bubbles correspond to the index of studies in the Appendix.

The meta-regression between the total number of sessions (*N* = 16) and the alleviation in craving was not significant [*Q*(1) = 0.0006, *p* = 0.98], so was the number of pulses in rTMS studies [*N* = 12, *Q*(1) = 0.37, *p* = 0.54]. However, we observed a negative relationship between the number of pulses per session and the rTMS outcome [*N* = 12, *Q*(1) = 8.04, *p* = 0.005] (Figure 4), while the overall days of stimulation [*N* = 16, *Q*(1) = 0.02, *p* = 0.88] and the number of sessions per day [*N* = 16, *Q*(1) = 0.60, *p* = 0.44] did not reveal significant effect.

## Discussion

The current study has confirmed the feasibility of using NIBS to allay cocaine or methamphetamine craving given a large main effect of stimulation (Hedge's *g* = 1.116). While, this effect showed heterogeneity that partly originated from subject variables which is consistent with the ubiquitous individual difference of NIBS effect (Ridding and Ziemann, 2010; López-Alonso et al., 2014). In addition, only high-frequency rTMS could elicit a significant treatment effect while the outcome of low-frequency rTMS was relatively controversial. We also found that the less pulses per session, the larger the NIBS effect would be. These results extended the notion toward NIBS intervention in multiple aspects.

In contrast to the current conclusion, previous clinical guidelines suggested that NIBS might not be applicable in the treatment of SUD (Lefaucheur et al., 2014, 2017). Such difference might stem from two aspects: first, the guidelines are based on qualitative integrations toward previous findings, while we quantitatively assessed the effect by calculating the overall effect size, by adding many recent findings (most of them are published after the year 2017). Meanwhile, other quantitative meta-analyses like Jansen et al. (2013) and Song et al. (2019) found significant main effect of NIBS treatment for SUD as well. On the other hand, we specifically probed the effect of NIBS over DA-dependent SUD, while the guidelines combined different kinds of SUD together. Thus, the inconsistency in the final outcomes might originate from the difference in the mechanisms of addiction among different SUDs.

A great many studies have effectively proved that NIBS could induce changes in cortical excitability (Ridding and Rothwell, 2007; Barr et al., 2008). A pulsatile electromagnetic field around the coil or direct current from the patch can induce an immediate excitatory effect to the neurons beneath the coil or patch (Spagnolo and Goldman, 2017). rTMS targeting at prefrontal areas could impact executive control functions (Stürmer et al., 2007). On the other hand, fronto-parietal circuit dysfunction has been found in stimulant abusers along with resultant deficits in executive functions (Goldstein and Volkow, 2002). Given that the current meta-analysis has revealed a frequency-specific pattern of rTMS treatment, high-frequency stimulation to the scalp may potentially produce long-term-potentiation-like (LTP-like) effects in the target cortical areas in a frequency-related manner (Ridding and Rothwell, 2007). Furthermore, 10-Hz rTMS to the prefrontal regions has been proved to induce changes in DA binding in monosynaptic striatal targets and the downstream frontal cortices (Strafella et al., 2001; Pogarell et al., 2006; Cho and Strafella, 2009). The increase in DA level elicited by rTMS was close to the aftereffect of amphetamine injection (Pogarell et al., 2007). Besides, cocaine and methamphetamine are substances that directly act on DA receptors. Our meta-analysis has ascertained the effectiveness of NIBS in alleviating craving to these two DA-dependent addictions which implied that NIBS treatment might alter DA-related functions. Take all these evidences into consideration, the DA theory of NIBS (Diana, 2011), which assumed that NIBS could antagonize the DA shortage in abusers through the upward spiral of PFC-VTA-NAc circuit (Figure 1), should be a tenable explanation to the observed treatment effect. Nonetheless, this hypothesis is still in lack of direct evidence so far. The causal link between the ramping up of DA level caused by NIBS and the alleviation in craving requires further test.

The current study also revealed that scaling up the number of pulses per session rather than the aggregate of pulses could induce harmness to the treatment. This implied that rTMS treatment should be provided in multiple sessions with each session ideally compressed. Stimulants like cocaine and methamphetamine manipulate DA level by physically altering DA receptor functions and gradually lead to desensitization to the external stimuli (Kahlig and Galli, 2003; Volkow et al., 2008; Wang et al., 2012). According to the DA theory of NIBS, rTMS could activate Dopaminergic neurons in VTA through the feedback projections from PFC and elicit DA release in striatal targets (Cho and Strafella, 2009; Diana, 2011). Hence, the negative moderation of the number of pulses per session might possibly stem from the desensitization of neurons in DA system or the brain regions beneath the coil induced by the intensive stimulation. As a result, there's expected to be a saturation point in the rTMS dose-response relationship after a certain number of pulses. Such saturation effects with pulses of over-dosage in SUD need to be further carefully considered, and generated to other applications of treatment with NIBS, such as depression or Parkinson's disease (Chou et al., 2015; Sehatzadeh et al., 2019), which also recruit the PFC-VTA DA pathway.

It should also be noted that Song et al. (2019) find a monotonic positive moderation effect of the number of pulses which is inconsistent with the current result. We argue that this might originate from the different ways of data extraction. The current analysis only used the result of the first probe after the stimulation in each study as the main effect size while Song et al. (2019) averaged all the craving scores in the post-stimulation probes, which could introduce the confounding factor of the relapse effect. Besides, Song et al. (2019) included the treatments of eating disorder and obesity in their analysis and they could have different dose-response properties compared with DA-drugs. Previous clinical guidelines regarding NIBS all focused on the stimulation parameters such as montages, frequency and intensity (Lefaucheur et al., 2014, 2017). However, to our knowledge, none of them attended to the methodology of segmentation. We believe that more future studies are needed to explore the prospective turning point in each session of treatment in order to optimize the stimulating protocol.

Despite the promising findings, the current meta-analysis had several limitations. First, there were only 12 studies survived by the screening, which led to a deficiency in statistical power. Specifically, only four units of tDCS trials were included in the analysis, so it would be premature to make conclusions regarding whether tDCS is useful in helping rehabilitation of SUD although three of the included units all showed positive effect (Shahbabaie et al., 2014, 2018; Batista et al., 2015). Further work is required to confirm the effect of tDCS in the light of its conspicuous convenience and cost-effectiveness. Second, our analysis could not reliably estimate the effect of stimulating regions other than the left DLPFC. Frontal-limbic loop has two separate sub-circuits. Executive control loop consists of DLPFC and dorsal striatum while limbic control loop comprises medial PFC (mPFC), ACC, and ventral striatum (Alexander et al., 1986). Martinez et al. (2018) employ H-coil to stimulate mPFC and ACC in cocaine-dependents. They find significant reduction in craving for the stimulating group after the 13-day high-frequency rTMS while their craving level does not differ from the sham group. However, Hanlon et al. (2015) detect that the decrease in craving for the stimulating condition is larger than the sham condition after a single-session cTBS targeting at the frontal pole in order to stimulate the ventral mPFC. Nonetheless, they do not replicate this effect in a recent study (Hanlon et al., 2017). Thus, the effect of stimulating cortices involved in the limbic control loop is still in controversy. Third, the current study is insufficient to test the follow-up effect. Although some studies have probed craving levels several days after the treatment, not all of them have reported the between-group difference in the relapse rate of craving level. Moreover, the interval between the end of treatment and the follow-up test was chosen inconsistently across those studies. Systematic investigations toward the temporal properties of NIBS effect in reducing craving would be informative in the future. Fourth, the current study should only be treated as a preliminary discussion about the mechanism of NIBS treatment. As a matter of fact, we still could not tell the origin of the rehabilitation: does it come from the direct alteration of excitability in the target cortices induced by stimulation, or through the mediation of Dopaminergic deep brain nuclei, or a mixture of the two candidate mechanisms? We believe that neuroimaging or lesion studies would be especially helpful in this issue.

Altogether, the current study indicated that NIBS is a safe and effective treatment for DA-dependent SUD. The heterogeneity in the previous trials comes from individual differences and the discrepancies in stimulation protocol. Future extensions should focus on the optimization of this promising technique by qualifying the current findings and meanwhile exploring the underlying mechanism in order to find a reliable and powerful treatment against SUD.

## Data Availability Statement

The original data of all the results could be found in the Supplementary Material.

## Author Contributions

YK: conceptualization, supervision, writing—review, editing, and funding acquisition. TM and YS: data curation and writing—original draft. TM: data analysis.

### Conflict of Interest

The authors declare that the research was conducted in the absence of any commercial or financial relationships that could be construed as a potential conflict of interest.
