# Supplementary material for: Effects of Non-invasive Brain Stimulation on Stimulant Craving in Users of Cocaine, Amphetamine, or Methamphetamine: A Systematic Review and Meta-Analysis
Source: Front Neurosci. 2019 Oct 18;13:1095. doi: 10.3389/fnins.2019.01095 (PMC6813242; doi:10.3389/fnins.2019.01095)
Supplement: Supplementary file 1 [file Data_Sheet_1.docx]

**Appendix**

| **Index** | **Study** | **Journal** | **Type of addiction** | **Stimulation Technique** | **Sample Size** | **Craving Indicator** | **Stimulation Site** | **Frequency** | **TMS Intensity** | **Days** | **Sessions** | **TMS Pulses** |
| --- | --- | --- | --- | --- | --- | --- | --- | --- | --- | --- | --- | --- |
| 1 | Li et al., (2013) | Drug & Alcohol Dependence | methamphetamine | rTMS | 10 | VAS | L DLPFC | 1 | 100% | 1 | 1 | 900 |
| 2 | Martinez et al., (2018) | Frontiers in Psychiatry | cocaine | rTMS | 12 | Number of choices | L&R ACC&mPFC | 1 | 110% | 21 | 13 | 11700 |
| 3 | Hanlon et al., (2017) | Drug & Alcohol Dependence | cocaine | cTBS | 25 | Self-report likert scale | L Frontal Pole | -- | 110% | 1 | 1 | 3600 |
| 4 | Liu et al., (2019) | Progress in Neuropsychopharmacology & Biological Psychiatry | methamphetamine | rTMS | 52 | VAS | L DLPFC | 10 | 100% | 20 | 20 | 40000 |
| 5 | Klauss et al., (2018) | Frontiers in Pharmacology | cocaine | tDCS | 33 | OCDS | L&R DLPFC | -- | -- | 19 | 10 | -- |
| 6 | Su et al., (2017) | Drug & Alcohol Dependence | methamphetamine | rTMS | 30 | VAS | L DLPFC | 10 | 80% | 1 | 1 | 6000 |
| 7 | Batista et al., (2015) | International Journal of Neuropsychopharmacology | cocaine | tDCS | 36 | OCDS | L&R DLPFC | -- | -- | 9 | 5 | -- |
| 8 | Martinez et al., (2018) | Frontiers in Psychiatry | cocaine | rTMS | 12 | Number of choices | L&R ACC&mPFC | 10 | 110% | 20 | 13 | 15600 |
| 9 | Shahbabaie et al., )2018) | Brain & Behavior | methamphetamine | tDCS | 15 | VAS | L&R DLPFC | -- | -- | 1 | 1 | -- |
| 10 | Bolloni et al., (2016) | Frontiers in Psychiatry | cocaine | rTMS | 10 | Hair Analysis | L&R PFC | 10 | 100% | 28 | 12 | 12000 |
| 11 | Shahbabaie et al., (2014) | International Journal of Neuropsychopharmacology | methamphetamine | tDCS | 30 | Self-report likert scale | R DLPFC | -- | -- | 1 | 1 | -- |
| 12 | Liu et al., (2017) | The American Journal on Addictions | methamphetamine | rTMS | 20 | Self-report likert scale | L DLPFC | 10 | 100% | 5 | 5 | 10000 |
| 13 | Liu et al., (2017) | The American Journal on Addictions | methamphetamine | rTMS | 20 | Self-report likert scale | R DLPFC | 10 | 100% | 5 | 5 | 10000 |
| 14 | Liu et al., (2017) | The American Journal on Addictions | methamphetamine | rTMS | 20 | Self-report likert scale | R DLPFC | 1 | 100% | 5 | 5 | 3000 |
| 15 | Liu et al., (2017) | The American Journal on Addictions | methamphetamine | rTMS | 20 | Self-report likert scale | L DLPFC | 1 | 100% | 5 | 5 | 3000 |
| 16 | Liang et al., (2018) | JAMA Psychiatry | methamphetamine | rTMS | 48 | Self-report likert scale | L DLPFC | 10 | 100% | 12 | 10 | 24000 |

**Table A1 | Detailed information of the included units of analysis.** VAS=Visual Analogue Scale, OCDS=Obsessive Compulsive Drinking Scale, ACC=anterior cingulate cortex, mPFC=medial prefrontal cortex, DLPFC=dorsolateral prefrontal cortex
